# Maternal Breast Milk Secretor Phenotype Does Not Affect Infant Susceptibility to Rotavirus Diarrhea

**DOI:** 10.1093/ofid/ofad299

**Published:** 2023-06-07

**Authors:** Frank B Williams, Md Abdul Kader, Dorothy M Dickson, E Ross Colgate, Masud Alam, Rashidul Haque, William A Petri, Beth D Kirkpatrick, Benjamin Lee

**Affiliations:** Vaccine Testing Center and Translational Global Infectious Diseases Research Center, Larner College of Medicine, University of Vermont, Burlington, Vermont, USA; International Centre for Diarrhoeal Disease Research, Bangladesh, Dhaka, Bangladesh; Vaccine Testing Center and Translational Global Infectious Diseases Research Center, Larner College of Medicine, University of Vermont, Burlington, Vermont, USA; Vaccine Testing Center and Translational Global Infectious Diseases Research Center, Larner College of Medicine, University of Vermont, Burlington, Vermont, USA; International Centre for Diarrhoeal Disease Research, Bangladesh, Dhaka, Bangladesh; International Centre for Diarrhoeal Disease Research, Bangladesh, Dhaka, Bangladesh; Division of Infectious Diseases and International Health, University of Virginia, Charlottesville, Virginia, USA; Vaccine Testing Center and Translational Global Infectious Diseases Research Center, Larner College of Medicine, University of Vermont, Burlington, Vermont, USA; Vaccine Testing Center and Translational Global Infectious Diseases Research Center, Larner College of Medicine, University of Vermont, Burlington, Vermont, USA

**Keywords:** breast milk, oral vaccination, rotavirus, secretor status

## Abstract

Breast milk secretor status is associated with antibody seroconversion to oral rotavirus vaccination. Here, we were unable to detect a similar impact on risk of infant rotavirus diarrhea or vaccine efficacy through 2 years of life, underscoring limitations of immunogenicity assessment alone in evaluation of oral rotavirus vaccine response.

Human rotavirus (RV) infections remain the leading global cause of infectious diarrhea in young children [1, 2]. Live-attenuated oral rotavirus vaccines have dramatically impacted disease burden but, for multiple reasons, underperform in low- to middle-income countries relative to high-income countries [3]. One potential variable is secretor status, a genetic determinant of RV host susceptibility. Secretor status is determined by an α[1,2]-fucosyltransferase encoded by the *FUT2* gene: individuals with functional enzyme are termed secretors and express specific 2-fucosylated oligosaccharides on mucosal surfaces and bodily secretions, including on the gut mucosa and in breast milk in the form of human milk oligosaccharides (HMOs). Nonsecretors lack functional enzyme and express a different glycan array in these body compartments.

Previously, we demonstrated that unvaccinated nonsecretor infants in Bangladesh had significantly reduced risk for RV diarrhea compared to secretors [4]. Furthermore, following oral vaccination with Rotarix (GlaxoSmithKline), Bangladeshi infants born to nonsecretor mothers had a significantly increased rate of plasma RV-specific immunoglobulin A (RV-IgA) seroconversion compared to infants born to secretor mothers, possibly due to interference in vaccine take from secretor-dependent antigens in breast milk [5]. However, whether maternal secretor status impacted protection from RV diarrhea remained unclear. Therefore, we expanded our previous post hoc analysis to include all evaluable mother–infant dyads to investigate the role of maternal secretor status on RV diarrhea. We hypothesized that maternal nonsecretor status would be associated with a significant difference in risk of RV diarrhea according to infant vaccination status.

## METHODS

We evaluated mother–infant dyads participating in the Performance of Rotarivus and Oral Polio Vaccines in Developing Countries (PROVIDE) study, a birth cohort study performed in Dhaka, Bangladesh, from 2011 to 2014 that included a Rotarix vaccine efficacy trial. PROVIDE was approved by the ethical review boards of the International Centre for Diarrhoeal Disease Research, Bangladesh, the University of Virginia, and the University of Vermont, and was registered at ClinicalTrials.gov (NCT01375647). All participating families provided signed informed consent. Infants were enrolled in the first week of life and randomized 1:1 to receive Rotarix at 10 and 17 weeks or to receive no vaccine. All infants were followed prospectively with active community diarrheal surveillance through 2 years of life.

RV diarrhea was diagnosed in diarrheal specimens by stool RV antigen detection using the ProSpecT enzyme immunoassay (EIA) kit (Oxoid, Hampshire, United Kingdom). RV-IgA and RV–immunoglobulin G (IgG) (in a subset of participants) were measured in plasma at 6 and 18 weeks of life by EIA; seropositive was defined as ≥20 U/mL, and seroconversion was defined as seronegative at week 6 for RV-IgA converting to seropositive at week 18 [6, 7]. Antibody concentrations were log-transformed for analysis. Secretor phenotype can be inferred from Lewis antigen status or directly measured using *Ulex europaeus* agglutinin 1 (UEA-1) EIA in Lewis antigen–negative individuals. Infant secretor phenotype was determined by saliva Lewis antigen dot-blot assay and UEA-1 EIA, and maternal breast milk phenotype was determined by Lewis a and b antigen EIA, with nonsecretors defined as Lewis a^+^/b^–^ and secretors as a^+^/b^+^ or a^–^/b^+^, as previously described [4, 5]. We were unable to validate the accuracy of UEA-1 EIA in breast milk specimens; therefore, breast milk analysis was limited to Lewis antigen–positive mothers.

Dichotomous variables were analyzed by χ^2^ test. Continuous variables were analyzed by independent samples *t* test. Due to the limited sample size of available RV-IgG measurements, we compared antibody concentrations according to maternal secretor status using all available data from the per-protocol population, irrespective of data completeness, in all children without rotavirus diarrhea by week 6 antibody measurement. Multivariable logistic regression was performed to assess the contribution of maternal secretor phenotype to incidence of RV diarrhea through year 1 and year 2 of life in the per-protocol population with complete data, controlling for multiple covariates previously determined to influence risk of RV diarrhea: RV-IgA seroconversion, infant secretor status, water treatment, weeks of exclusive breastfeeding, week 18 zinc concentration, and week 10 length-for-age *z* score, with calculation of odds ratios (ORs) and associated 95% confidence intervals (CIs) [4]. Because we previously showed that infant secretor phenotype interacted with vaccination status, separate models were fitted according to infant vaccination status. Based on our previous findings, we hypothesized that the effect of maternal secretor phenotype would be mediated by RV-IgA seroconversion. To test this, we compared a controlled effect model including all listed covariates to a total effect model, in which RV-IgA seroconversion was excluded to assess the overall contribution of maternal secretor phenotype independent of RV-IgA seroconversion. Additional models were fit as above including week 6 RV-IgG (ie, maternal antibodies). Vaccine efficacy (VE) through 1 and 2 years was calculated as [(risk among unvaccinated – risk among vaccinated) / risk among unvaccinated]. Because the postvaccination period was defined as 18–104 weeks, vaccinated infants with RV diarrhea prior to week 18 were excluded from analysis. All analyses were conducted using SPSS software version 27.0.0 (IBM, Armonk, New York) or GraphPad Prism software version 9.3.1 (GraphPad, La Jolla, California). Differences were considered statistically significant at 2-sided *P* < .05.

## RESULTS

A total of 486 mother–infant dyads had maternal breast milk secretor phenotype data available. Characteristics of the included study population are presented in Table 1. As expected, infant nonsecretors were more frequently born to maternal nonsecretors. In pooled analysis irrespective of vaccination status, the prevaccination maternal antibody level (geometric mean concentration) in infants of maternal secretors (n = 162) was 397.4 (95% CI, 429.6–479.3) compared to 203.9 (95% CI, 153.9–270.2) in infants of maternal nonsecretors (n = 73) (*P* = .0001).

**Table 1.** Participant Characteristics According to Rotarix Vaccination and Maternal Secretor Status

Characteristic	Unvaccinated Infants (n = 227)				Vaccinated Infants (n = 216)			
	Maternal Secretor (n = 156)	Maternal Nonsecretor (n = 71)	Total	*P* Value	Maternal Secretor (n = 148)	Maternal Nonsecretor (n = 68)	Total	*P* Value
Sex								
Male	87 (55.8)	33 (46.5)	120 (52.9)	.2	79 (53.4)	35 (52.8)	114 (52.8)	.8
Female	69 (44.2)	38 (53.5)	107 (47.1)		69 (46.6)	33 (47.2)	102 (47.2)	
Infant secretor status								
Secretor	113 (72.4)	37 (52.1)	150 (66.1)	.003	121 (81.8)	28 (41.2)	149 (69.0)	2 × 10^−9^
Nonsecretor	43 (27.6)	34 (47.9)	77 (33.9)		27 (18.2)	40 (58.8)	67 (31.0)	
Week 18 RV-IgA								
Seroconversion	31 (19.9)	13 (18.3)	44 (19.4)	.8	32 (21.6)	25 (36.8)	57 (26.4)	.019
No seroconversion	125 (80.1)	58 (81.7)	183 (80.6)		116 (78.4)	43 (63.2)	159 (73.6)	
GMC (95% CI)	13.4 (11.1–16.0)	12.3 (9.5–16.0)	NA	.61	18.3 (14.3–23.3)	21.6 (15.2–30.8)	NA	.4

Data are shown as No. (%) unless otherwise indicated.

Abbreviations: CI, confidence interval; GMC, geometric mean concentration; NA, not applicable; RV-IgA, plasma rotavirus-specific immunoglobulin A.

We then assessed the impact of maternal secretor breast milk phenotype on incidence of RV diarrhea among unvaccinated infants. Among 242 (49.8%) unvaccinated mother–infant dyads, 227 had complete data for analysis, of whom 156 (68.7%) were maternal secretor and 71 (31.3%) were maternal nonsecretor. In multivariable logistic regression, infant nonsecretor phenotype and weeks of exclusive breastfeeding were significantly associated with incidence of RV diarrhea through both 1 year and 2 years of life, but maternal secretor phenotype was not (Table 2). No significant interactions were noted between any of the tested covariates. These results suggest that maternal phenotype was not associated with risk of RV diarrhea in unvaccinated children.

**Table 2.** Multivariable Logistic Regression of Rotavirus Diarrhea Risk of Any Severity Through Year 1 and Year 2 of Life

Variable	Unvaccinated			Vaccinated		
	OR	(95% CI)	*P* Value	OR	(95% CI)	*P* Value
Through year 1						
Infant nonsecretor phenotype	0.335	(.173–.648)	.001	0.571	(.223–1.460)	.242
Lack of RV-IgA seroconversion	1.302	(.611–2.773)	.494	2.73	(.983–7.581)	.054
Lack of water treatment	2.008	(1.104–3.651)	.022	1.06	(.491–2.287)	.883
HAZ at week 10	0.913	(.668–1.248)	.568	1.058	(.679–1.648)	.804
Serum zinc level at week 18	1.018	(.996–1.041)	.104	1.044	(1.012–1.078)	.007
Weeks of exclusive breastfeeding	1.045	(1.009–1.083)	.013	0.999	(.958–1.042)	.966
Maternal nonsecretor phenotype	1.332	(.699–2.540)	.384	0.943	(.376–2.367)	.901
Through year 2						
Infant nonsecretor phenotype	0.406	(.223–.739)	.003	0.439	(.201–.962)	.04
Lack of RV-IgA seroconversion	1.567	(.764–3.213)	.220	3.366	(1.484–7.634)	.004
Lack of water treatment	1.503	(.842–2.682)	.168	0.917	(.477–1.765)	.795
HAZ at week 10	0.978	(.726–1.318)	.885	1.113	(.772–1.603)	.567
Serum zinc level at week 18	1.019	(.998–1.040)	.075	1.036	(1.011–1.062)	.005
Weeks of exclusive breastfeeding	1.045	(1.011–1.082)	.01	0.984	(.949–1.019)	.36
Maternal nonsecretor phenotype	0.858	(.465–1.583)	.625	1.406	(.658–3.005)	.38

Abbreviations: CI, confidence interval; HAZ, height-for-age *z* score; OR, odds ratio; RV-IgA, plasma rotavirus-specific immunoglobulin A.

Next, we analyzed vaccinated infants. Among 244 (50.2%) vaccinated mother–infant dyads, 216 had complete data for analysis, of whom 148 (68.5%) were maternal secretors and 68 (31.5%) were maternal nonsecretors. In multivariable logistic regression, zinc level was associated with RV diarrhea incidence through year 1, but infant and maternal secretor phenotypes were not (Table 2). No notable differences were observed when RV-IgA seroconversion was excluded from the model, suggesting that the effect of maternal secretor phenotype RV diarrhea risk was unrelated to RV-IgA seroconversion (data not shown). Through year 2, zinc level, lack of RV-IgA seroconversion, and infant nonsecretor phenotype were significantly associated with RV diarrhea, but maternal phenotype was not (Table 2). When RV-IgA seroconversion was excluded, the strength of the effect of infant nonsecretor phenotype was attenuated (OR, 0.511 [95% CI, .242–1.080]; *P* = .079), but no other notable changes were observed (data not shown). No significant interactions were noted between any of the tested covariates.

In a smaller subset of infants with evaluable data (n = 107 unvaccinated, n = 112 vaccinated), inclusion of maternal antibody concentrations had no impact on any of the model outcomes described above (data not shown).

Finally, we evaluated the impact of maternal secretor status on VE. Overall VE against RV diarrhea of any severity through year 1 of life was 47.0% (95% CI, 34.3%–69.3%), similar to VE through year 1 for the parent cohort (51%) [6]. VE against RV diarrhea through year 1 of life was 44.8% (95% CI, 25.4%–69.6%) among infants of maternal secretors and 53.0% (95% CI, 15.1%–83.3%) among infants of maternal nonsecretors, a difference of +8.2% in infants of nonsecretors. However, this difference was not sustained through year 2. VE through year 2 was 34.0% (95% CI, 25.4%–59.9%), lower than previously reported for RV-naive children through year 2 for the parent cohort (48.2%) [8]. VE against RV diarrhea of any severity through year 2 of life was 37.6% (95% CI, 28.2%–71.9%) among infants of maternal secretors and was 25.2% (95% CI, −28.3% to 67.5%) among infants of maternal nonsecretors, a difference of −12.4% in infants of maternal nonsecretors.

## DISCUSSION

Maternal secretor phenotype was not associated with risk of RV diarrhea through year 1 or year 2 of life among children in Dhaka, Bangladesh, and had no appreciable effect on Rotarix VE. Controlling for maternal secretor phenotype had no impact on covariates previously shown to be important in modulating RV diarrhea risk in this population, with the exception of higher serum zinc levels associated with risk of RV diarrhea among vaccinated infants, contrary to previous findings [4–6]. These data are consistent with prior data from select MAL-ED (Malnutrition and Enteric Disease study) sites showing no significant differences in infant RV diarrhea risk based on maternal secretor status [9].

In a larger sample size of week 6 RV-IgG antibody measurements (ie, transplacentally acquired maternal antibodies), RV-IgG in infants of maternal secretors was significantly higher than in infants of nonsecretors, consistent with findings in adults [10]. Due to limitations in available sample size, we failed to detect this difference previously when assessing only vaccinated children with complete data [5], and it is indeed highly plausible that differences in vaccine immunogenicity based on maternal status may be driven primarily by this effect on maternal antibody levels [11]. Despite this, neither maternal status nor maternal antibody concentration was significantly associated with risk of RV diarrhea in either vaccinated or unvaccinated children when controlling for other pertinent variables, although results should be interpreted with caution, again due to small sample sizes.

Our results thus further highlight inherent limitations of using immunogenicity endpoints alone when assessing oral vaccine responses. There are several possible reasons why maternal secretor phenotype did not appreciably affect VE, despite previous data from this cohort demonstrating a significant effect of maternal phenotype on postvaccination RV-IgA seroconversion [5]. Since RV-IgA appears to explain a fraction of the overall vaccine effect [7, 12] and maternal phenotype improved seroconversion only among the minority of infants born to maternal nonsecretors, the overall contribution of maternal phenotype to VE may be limited in scope. In addition, following the period of exclusive breastfeeding, any incremental impact of maternal status is likely far outweighed by the profound effect of infant phenotype on RV susceptibility. Limitations of this study include the post hoc nature of this subset analysis, which limited sample size and power and may have introduced selection bias. Because our regression analysis required stratification according to vaccination status, results are not directly comparable to the approach used in the primary efficacy evaluation for the parent cohort. Since HMO concentrations vary over time, phenotype misclassification is a risk. While maternal genotype provided the strongest predictor of infant vaccine seroconversion, too few mothers had available DNA for genotyping for meaningful RV diarrhea analysis.

Numerous studies have confirmed the importance of secretor status on risk of RV infection and diarrhea. While our results suggest that maternal status may not significantly impact Rotarix VE, evaluation of oral RV vaccine response provides a useful model for the study of susceptibility to RV infection in children. More definitive, adequately powered longitudinal studies accounting for maternal secretor status, maternal antibodies, and HMO composition over the course of infancy in relation to RV vaccine efficacy and diarrhea susceptibility are needed.

## Notes

***Financial support*.** This work was supported by the National Institute of General Medical Science at the National Institutes of Health (grant number P20 GM125498 to B. D. K. and B. L.); the National Institutes of Health (grant number R01 AI043596 to W. A. P.); the Bill & Melinda Gates Foundation (grant number OPP1127782 to B. D. K.); the Foundation for the Society for Maternal-Fetal Medicine (Queenan Fellowship for Global Health to F. B. W.); and the International Centre for Diarrhoeal Disease Research, Bangladesh (the governments of Bangladesh, Canada, Sweden, and the United Kingdom (core/unrestricted support)).
